# Nitric Oxide-Releasing *S*-Nitrosoglutathione-Conjugated TEMPO-Oxidized Nanocellulose Hydrogel for the Treatment of MRSA-Infected Wounds

**DOI:** 10.3390/pharmaceutics17121623

**Published:** 2025-12-17

**Authors:** Dongmin Kwak, Chavi Dagar, Jihyun Kim, Juho Lee, Hyunwoo Kim, Muneeb Ullah, Md. Lukman Hakim, Minjeong Kim, Mst. Sanzida Yeasmin, Ng’wisho Nyalali, Jin-Wook Yoo

**Affiliations:** 1 College of Pharmacy, Pusan National University, Busandaehak-ro 63 beon-gil 2, Geoumjeong-gu, Busan 46241, Republic of Korea; pharm0701@gmail.com (D.K.); chavidagaredu@gmail.com (C.D.); jihyun@pusan.ac.kr (J.K.); rlagusdn0628@naver.com (H.K.); munibdawar72@gmail.com (M.U.); mlhshohag1221@gmail.com (M.L.H.); kminjeong0910@gmail.com (M.K.); yeasminsanzida03@gmail.com (M.S.Y.); dnyalali@gmail.com (N.N.); 2 Research Institute for Drug Development, Pusan National University, Busan 46241, Republic of Korea; 3 College of Pharmacy, Daegu Catholic University, Gyeongsan 38430, Republic of Korea; leejh235@cu.ac.kr

**Keywords:** *S*-nitrosoglutathione, MRSA, nanocellulose, nitric oxide, conjugation

## Abstract

**Background**: Cutaneous wound infections caused by methicillin-resistant *Staphylococcus aureus* (MRSA) pose serious threats to public health. Nitric oxide (NO), an endogenous gaseous molecule with antibacterial and wound-healing properties, is a promising next-generation antimicrobial agent with a minimal risk of resistance. However, conventional *S*-nitrosoglutathione (GSNO)-loaded formulations suffer from GSNO leakage, which could compromise the treatment effect or induce systemic side effects. Although conjugation strategies have been introduced to mitigate this issue, there is still a lack of GSNO-conjugated systems that simultaneously achieve high NO loading and sustained NO release while avoiding harsh external stimuli and complex multistep synthetic processes. **Objectives**: This research aims to develop a high NO-loading system produced through a simple synthetic process that provides sustained NO release without harsh external stimuli while preventing GSNO leakage for effective treatment of MRSA-infected wounds. **Methods**: We developed cellulose-based GSNO conjugates via a simple EDC/NHS-mediated covalent coupling to TEMPO-oxidized nanocellulose (NC-GSNO). **Results**: The NC-GSNO hydrogel achieved high NO loading, minimal leakage, and sustained NO release for more than three days. This controlled NO delivery promoted enhanced wound healing in MRSA-infected models. **Conclusions**: These findings demonstrate that the NC-GSNO hydrogel is a promising platform for controlled NO delivery and the effective treatment of MRSA-infected wounds.

## 1. Introduction

Infections of cutaneous wounds with methicillin-resistant *Staphylococcus aureus* (MRSA), one of the major pathogens in skin and soft tissue infections, pose a formidable threat to public health [1,2]. For the efficient treatment of MRSA-infected wounds, eradicating the bacteria at the wound site is the primary goal because bacterial infections at the wound site can delay wound closure owing to serious inflammation [3]. This inflammation not only destroys normal wound tissue through elevated reactive oxygen species levels and proteinase activities but also hampers tissue regeneration by increasing the signaling of pro-inflammatory cytokines [4]. This prolonged open-wound state increases the risks of serious complications, such as permanent scarring, amputation, systemic infection, and death [5,6]. The increase in antibacterial resistance has rendered treating infections using traditional antibiotics, such as vancomycin, difficult [7]. Therefore, next-generation antimicrobial agents with minimal bacterial resistance are urgently required.

Nitric oxide (NO), an endogenous gaseous molecule with various functions in physiological systems, is known for its antibacterial properties [8]. NO disrupts the homeostasis of bacterial, viral, and fungal physiological systems through the nitrosation of proteins and the oxidation of nucleic acids and cell membranes, which ultimately leads to microbial death [9]. This multi-target mechanism makes NO a promising agent that poses a low risk of inducing bacterial resistance [10]. In addition, NO promotes wound healing by promoting the proliferation of keratinocytes, fibroblasts, and endothelial cells [11,12]. These characteristics make NO a promising candidate for the healing of MRSA-infected wounds. However, because of its gaseous nature and short half-life, appropriate NO donors are required for the use of NO in therapeutic agents. Various NO donors—including diazeniumdiolates (NONOates), metal nitrosyls, and *S*-nitrosothiols (RSNOs) such as *S*-nitroso-*N*-acetylpenicillamine (SNAP) and *S*-nitrosoglutathione (GSNO)—have been widely studied for their NO-releasing properties [13]. Among them, GSNO stands out as one of the most biocompatible NO donors due to its endogenous origin and its ability to release NO under mild physiological conditions [14,15]. To maximize the therapeutic potential of GSNO, it is crucial to continuously provide NO to the target site, as NO exhibits strong concentration- and time-dependent antibacterial and wound-healing effects [16]. However, the direct application of GSNO to infected wounds is significantly limited, as the open wound structure and exposed blood vessels allow this highly hydrophilic, low-molecular-weight molecule to rapidly diffuse away from the site. Such leakage can lead to systemic side effects, including a sudden drop in blood pressure [17,18]. Additionally, the intrinsic chemical lability of GSNO can lead to burst NO release. Together, these factors reduce continuous local NO exposure and thus compromise its antibacterial and wound healing effects [18]. Accordingly, optimized GSNO formulations are necessary to ensure continuous NO exposure at the wound site to fully exert its potential. As part of these efforts, various physically GSNO-loaded formulations have been developed, including films and hydrogels [19,20,21,22,23,24]. Kim et al. [20] reported a GSNO-loaded chitosan film for infected wounds, while Razmjooee et al. [24] described a GSNO-loaded carboxymethyl chitosan–alginate hydrogel for diabetic wounds. These studies demonstrated enhanced wound healing outcomes. Nevertheless, since GSNO is incorporated physically rather than chemically, these systems remain inherently susceptible to undesirable GSNO leakage.

Building on the limitation in physically GSNO-loaded formulations, several studies have reported GSNO-conjugated systems for wound healing. Lee et al. [25] developed poly(lactic-co-glycolic acid) (PLGA)–GSNO-conjugated nanoparticles using an O/W emulsion. This study represented an early attempt to incorporate hydrophilic GSNO into hydrophobic PLGA nanoparticles via covalent conjugation, but the NO loading capacity was low (0.07 µmol/mg), and most of the loaded NO (~99%) was released within 12 h, indicating limited stability in the release. Catori et al. [26] reported a PLGA–GSNO-conjugated nanoparticle-loaded hydrogel system in which UV irradiation was used during the hydrogel crosslinking process. Although their system exhibited sustained NO release, the use of UV irradiation during crosslinking could pose a risk of tissue damage, and the lack of in vivo evaluation makes it difficult to assess its therapeutic efficacy. Wang et al. [27] recently reported a polydopamine–GSNO-conjugated nanoparticle system incorporated into a hydrogel to achieve photothermally triggered NO release under near-infrared irradiation. Although these systems enabled the external control of NO release, the high temperatures required (>50 °C) could cause tissue damage, limiting their suitability for practical wound healing applications. Taken together, these studies highlight that, although chemical conjugation can reduce GSNO leakage, there is still a lack of GSNO-conjugated systems that simultaneously achieve high NO loading and sustained NO release, while also avoiding the use of harsh external stimuli. Moreover, the multistep and harsh synthetic procedures involved in these systems may limit their reproducibility. Therefore, a novel system that simultaneously addresses these limitations is required.

**Figure 1:**
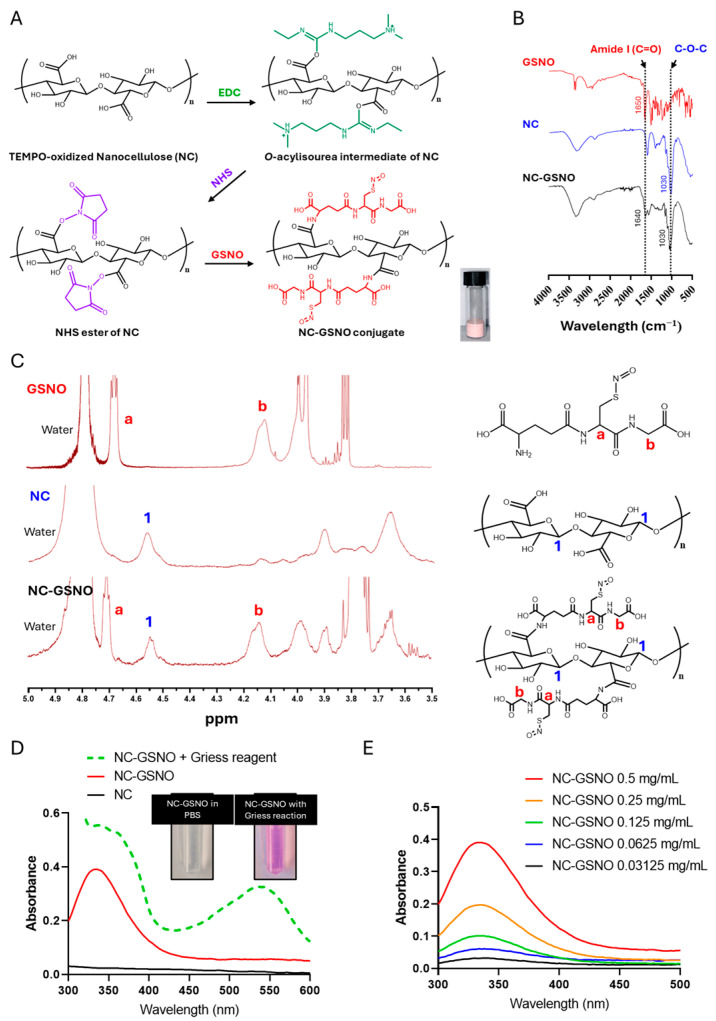
Characterization of NC-GSNO: (**A**) Synthesis procedure of NC-GSNO. (**B**) FTIR analysis of GSNO, NC, and NC-GSNO. (**C**) ^1^H-NMR analysis of GSNO, NC, and NC-GSNO (D_2_O, 600 MHz). (**D**) UV–Vis spectra of NC-GSNO after the Griess reaction. (**E**) UV–Vis spectra of various concentrations of NC-GSNO.

Cellulose nanofibers obtained by TEMPO-oxidation (TEMPO-oxidized nanocellulose, NC) are fibrous cellulose composed of linear glucose chains linked by β-(1,4) glycosidic bonds, in which the C6 hydroxyl groups are oxidized to carboxylic acids (the repeating unit is shown in Figure 1A) [28]. NC exhibits exceptional biocompatibility, biodegradability, non-immunogenicity, and mechanical robustness [29]. In addition, its dense surface carboxyl and hydroxyl groups enable electrostatic and hydrogen-bonding interactions that form a hydrogel, allowing NC to exhibit retentive behavior at the wound site [30]. Furthermore, its functional groups provide versatile reactive sites for further modifications, such as primary amine conjugation via 1-ethyl-3-(3-dimethylaminopropyl)carbodiimide (EDC) and *N*-hydroxysuccinimide (NHS). Owing to these favorable properties, various NC-based formulations have been developed for wound healing and drug delivery [29,31].

Based on these advantages, we developed GSNO-conjugated TEMPO-oxidized NC (NC-GSNO) using a simple and efficient synthetic method. The abundant carboxyl groups of NC provide multiple conjugation sites for GSNO, enabling high NO loading. Additionally, the hydrophilic microenvironment stabilizes the *S*–nitroso bond (S–N=O), thereby minimizing rapid GSNO degradation and enabling sustained NO release without the use of harsh external stimuli [32,33,34,35]. The hydrophilic nature of NC further enhances its residence at the wound site and helps retain moisture, thereby facilitating prolonged GSNO presence. This conjugation strategy is expected to overcome the limitations of previous GSNO formulations, including GSNO leakage, unstable NO release, and dependency on external stimuli. We therefore propose the use of NC-GSNO hydrogels as promising platforms for controlled NO delivery and enhanced wound healing.

## 2. Materials and Methods

### 2.1. Materials

Cellulose nanofibers obtained from 2,2,6,6-Tetramethylpiperidine-1-oxyl (TEMPO) oxidation (TEMPO-oxidized nanocellulose, NC; Re:ancel T-CNF powder, powder form, diameter < 10 nm and aspect ratio ~1:100, according to the manufacturer) were purchased from ANPOLY (Seoul, Republic of Korea). EDC, NHS, and Dulbecco’s Modified Eagle Medium (DMEM) were purchased from Thermo Fisher Scientific (Waltham, MA, USA). Penicillin G sodium and streptomycin sulfate were purchased from Welgene (Gyeongsan, Republic of Korea). CCK-8 assay reagent was purchased from Dogenbio (Seoul, Republic of Korea). Acetone was purchased from Daejung Chemical & Metals Co., Ltd. (Siheung, Republic of Korea). Tegaderm™ film dressing and an 8 mm biopsy punch were purchased from 3M (St. Paul, MN, USA). Dialysis membrane (12,000 Da MWCO) and Griess assay reagent were purchased from Sigma-Aldrich (St. Louis, MO, USA). Deuterium oxide (D_2_O, 99.9% atom D) was purchased from Cambridge Isotope Laboratories (Tewksbury, MA, USA).

### 2.2. Synthesis of GSNO

GSNO was prepared as previously described [36]. In brief, glutathione was dissolved in 0.625 M HCl, and an equivalent molar of sodium nitrite was added to the glutathione solution at 300 rpm for 40 min in an ice bath. After the reaction, an acetone wash was performed twice, followed by ether washing and vacuum drying. Freeze drying was followed after filtration (0.22 µm) of GSNO solution and the resultant GSNO was kept at −20 °C for future use.

### 2.3. Preparation of NC-GSNO

After autoclaving the TEMPO-oxidized NC suspension (200 mg in 8 mL) at 121 °C for 15 min, 1 mL of NHS solution (200 mg/mL) and 1 mL of EDC solution (200 mg/mL), each passed through a 0.22 µm filter, were added and allowed to react for 1 h at RT and 500 rpm under aseptic conditions [37]. The reactant was washed twice with 50% acetone and centrifuged at 3000 *g* for 5 min. The obtained NC-NHS ester was dissolved in 10 mL of sterilized distilled water, and 200 mg of GSNO was added as a powder and allowed to react for 2 h at RT with stirring at 500 rpm. After the reaction, the product was dialyzed overnight (12,000 MWCO, 4 °C, sterilized distilled water), freeze-dried, and stored at −20 °C.

### 2.4. Loading of NC-GSNO

A Sievers Nitric Oxide Analyzer (NOA 280i, GE Analytical Instruments, Boulder, CO, USA) was used to determine the NO and GSNO loading capacity of NC-GSNO, following previously reported methods [38]. In brief, after establishing a standard curve (range: 0–3 µmol NO) using potassium iodide and sodium nitrite following the manufacturer’s protocol, 1 mg of NC-GSNO powder was added to a NO-releasing chamber containing 30 mM ascorbic acid. The chamber was degassed with Ar gas supplied at a flow rate of 80 mL/min. The sample’s area under the curve (AUC) was converted to the amount of NO (µmol) per 1 mg of NC-GSNO and subsequently expressed as NO loading (µmol/mg). The GSNO loading capacity was calculated using Equation (1), based on a 1:1 molar relationship between NO and GSNO, using a GSNO molecular weight of 336.32 g/mol:

(1) $$\begin{array}{c} \;GSNO\;loading\;(\%)=NO\;loading\;(\mu mol/mg)\times 336.32\;(g/mol)\times 100/1000 \end{array}$$

### 2.5. Characterization of NC-GSNO

Fourier-transform infrared (FTIR) spectra were recorded using a Nicolet^TM^ iS50 FTIR spectrometer (Thermo Fisher Scientific, Waltham, MA, USA). Dry powder for each sample was prepared and analyzed using the attenuated total reflectance method in the range of 4000–500 cm^−1^ and at a resolution of 4 cm^−1^.

A proton nuclear magnetic resonance (NMR) analysis was performed using the 600 MHz Agilent NMR System (Agilent Technologies, Santa Clara, CA, USA). Dry powders for each sample were prepared using D_2_O. Specifically, 15 mg of each sample powder was added to 1 mL of D_2_O and transferred to a glass NMR tube immediately before analysis to prevent premature NO release during preparation.

UV–Vis spectra were recorded using a Hitachi U-5100 spectrophotometer (Hitachi High-Tech Corporation, Tokyo, Japan). For the Griess assay, 0.5 mL of the Griess reagent solution and 0.5 mL of the NC-GSNO suspension were mixed and allowed to react at RT for 20 min, after which the absorbance spectrum was measured with background correction. Concentration-dependent profiles of the S–N=O bond were prepared and analyzed for various concentrations of NC-GSNO (0.5, 0.25, 0.125, 0.0625, and 0.03125 mg/mL) in phosphate-buffered saline (PBS, pH 7.4). For each concentration, a corresponding blank was prepared using NC-GSNO at the same concentration from which NO had been completely removed. Complete NO removal was verified using a Sievers Nitric Oxide Analyzer. The blanks were used for background correction during UV–Vis measurements, ensuring that only the absorbance from the S–N=O bond was analyzed.

Scanning electron microscopy (SEM) was performed using a GEMINI 500 instrument (Carl Zeiss, Oberkochen Germany). The hydrogels were dispersed onto a carbon tape, freeze-dried, air-blown to remove excess debris, sputter-coated with platinum, and analyzed at an intensity of 5 keV.

### 2.6. Gelation and Water Uptake

Gel formation (200 mg/mL) was evaluated using the vial inversion method at room temperature after 5 min of hydration with distilled water. The fully hydrated gel sample was carefully transferred into the vials using a pipette prior to the inversion test. Hydrogel water uptake was assessed after determining the dry weight of each sample. The samples were initially frozen at −20 °C and then freeze-dried using a lyophilizer (FDU-1200, EYELA, Tokyo, Japan) at −50 °C under a vacuum pressure of 0.01 mbar (1 Pa) for 72 h until a constant weight was achieved. After the dry weight was obtained, the samples were immediately immersed in water at room temperature. The swollen weight was measured at predetermined time points after carefully blotting excess surface water with a paper towel. The water uptake ratio was calculated using Equation (2), where *W* indicates the mass of the hydrogel [39]:

(2) $$\begin{array}{c} Water\;uptake\;(\%)=\left(\frac{W_{wet}{-W}_{dry}}{W_{dry}}\right)\times 100 \end{array}$$

### 2.7. GSNO Leakage

A Franz diffusion cell (PermeGear, Hellertown, PA, USA), consisting of a donor chamber and a receiver chamber (8 mL) filled with PBS (pH 7.4) was used for GSNO leakage analysis. The receiver chamber solution was stirred at 100 rpm, and the system was incubated at 37 °C throughout the experiment. As a filter membrane, 12,000 MWCO dialysis membrane was used (effective diffusion area: 1.0 cm^2^). The NO-loaded NC-GSNO (0.5 mL; 126 mg/mL) and an NC + GSNO mixture (0.5 mL; NC: 101 mg/mL, GSNO: 25 mg/mL) were prepared in PBS (pH 7.4) and transferred into the donor chamber using a pipette. At each predetermined time interval, 100 µL of the solution was sampled from the receiver chamber and replaced with an equal volume of fresh PBS (pH 7.4). Based on the method described by [40], samples were analyzed to detect the amount of leaked GSNO using a Shimadzu HPLC system (Shimadzu, Kyoto, Japan) equipped with a C18 reverse-phase column (4.6 × 250 mm, 5 µm; VDS optilab, Berlin, Germany), with 5% acetonitrile as the mobile phase at a flow rate of 0.5 mL/min, column temperature of 30 °C, and UV detection at 200 nm. Calibration curves for GSNO quantification were generated over a concentration range of 2–250 µg/mL, ensuring adequate sensitivity for the concentrations measured in this study.

### 2.8. NO Release of NC-GSNO

The NO release of NC–GSNO was monitored using a U-5100 UV–Vis spectrophotometer by measuring the absorbance at 335 nm, corresponding to the S–N=O bond [41]. NC–GSNO was prepared at 0.5 mg/mL in PBS (pH 7.4), transferred into a cuvette, and incubated at 37 °C. At predetermined time points, the absorbance at 335 nm was recorded and converted to the remaining GSNO concentration using a standard calibration curve (range: 30–700 µg/mL). Because GSNO releases NO through a 1:1 molar degradation pathway, cumulative NO release at time t (%) was calculated from the decrease in GSNO concentration using Equation (3):

(3) $$\begin{array}{c} Cumulative\;NO\;release\;at\;time\;t\;(\%)=\frac{[GSNO{]}_{0}-[GSNO{]}_{t}}{[GSNO{]}_{0}}\times 100 \end{array}$$

### 2.9. In Vitro Antibacterial Study

The antibacterial activity of NC-GSNO was evaluated against methicillin-resistant *Staphylococcus aureus* (MRSA; strain USA300 FPR3757, GenBank accession no.: NC_007793) under aseptic conditions. MRSA was cultured overnight in tryptic soy broth at 37 °C. After washing the MRSA suspension twice with fresh sterilized PBS (pH 7.4) and centrifuging it at 10,000× *g* for 10 min. Then, 100 µL of bacterial stock (OD_600_ = 0.5) was mixed with 100 µL of NC (final concentration of 50 mg/mL) or various concentrations of NC-GSNO (final concentration of 10, 25, and 50 mg/mL) containing PBS (pH 7.4) and incubated at 37 °C for 24 and 48 h. After each exposure, the treated bacterial suspensions were serially diluted and plated on tryptic soy agar to determine colony-forming units (CFUs).

To visualize the live and dead status of MRSA, bacterial samples treated with NC (50 mg/mL) or NC-GSNO (50 mg/mL) for 48 h were stained using the LIVE/DEAD^®^ BacLight^TM^ bacterial viability kit (Thermofisher Scientific, Waltham, MA, USA) composed of SYTO9 and propidium iodide (PI), following the manufacturer’s protocol.

### 2.10. In Vitro Cytotoxicity Study

Cytotoxicity of NC-GSNO was evaluated in vitro using CCK-8 assay under aseptic conditions [42]. L929 mouse fibroblasts (Korean Cell Line Bank, Seoul, Republic of Korea) were cultured in 96-well plates at a density of 1 × 10^4^ cell/well in 100 µL of DMEM supplemented with 10% (*v*/*v*) FBS and antibiotics (100 IU/mL of penicillin G sodium and 100 µg/mL of streptomycin sulfate). The cells were incubated at 37 °C with 5% CO_2_ for 24 h. Then, the culture medium was replaced with serum-free DMEM containing NC-GSNO with different concentrations (0, 1, 10, 25, and 50 mg/mL) and incubated for 24 h. The plate was washed with fresh pre-warmed PBS, and 100 µL of DMEM containing 10% (*v*/*v*) CCK-8 reagent was added to each well. After 2 h incubation at 37 °C, the absorbance at 450 nm was detected. The cell viability was calculated using Equation (4):

(4) $$\begin{array}{c} Cell\;viability\;(\%)=\frac{Absorbance\;\left(treated\;cells\right)}{Absorbance\;\left(control\;cells\right)}\times 100\; \end{array}$$

### 2.11. In Vitro Cell Migration Study

Cell migration was evaluated in vitro using the scratch assay under aseptic conditions. L929 mouse fibroblasts were cultured in 24-well plates until they reached complete confluence. A scratch was made across the cell monolayer using a sterile 1 mL pipette tip, followed by gentle washing with sterilized PBS (pH 7.4) to remove detached cells and debris. The cultures were then treated by placing 10 and 50 mg of NC-GSNO powder (final concentration of 10 and 50 mg/mL) in Transwell insert (0.4 µm pore size, SPL Life Sciences, Pocheon, Republic of Korea), while 1 mL of DMEM was added to the bottom compartment containing scratched cells. Images were captured at 0 and 24 h post treatment to monitor cell migration. For the quantification of cell migration, the width ($W_{t}$) at each time point was calculated. Cell migration was expressed as the percentage of wound closure using Equation (5):

(5) $$\begin{array}{c} Migration\;(\%)=\frac{W_{0}-W_{t}}{W_{0}}\times 100\; \end{array}$$

### 2.12. MRSA-Infected Wound Model

All animal experiments were approved by the Institutional Animal Care and Use Committee of Pusan National University (PNU-IACUC, approval number: PNU-2025-0441; approved on 15 March 2025). All animals were housed and maintained in a specific-pathogen-free (SPF) facility under standard laboratory conditions. Six-week-old male imprinted control region (ICR) mice were purchased from Samtako Bio Korea (Osan, Republic of Korea) and acclimatized for 3 days before inducing the full-thickness infection wound model. The mice were randomly divided into four groups (*n* = 5 per group). After anesthesia with 0.6 mg/g of avertin (2,2,2-tribromoethanol) via intraperitoneal injection, hair was removed using a trimmer and depilation cream, and an 8 mm biopsy was performed to create a full-thickness wound. After inoculating 10^7^ CFU of MRSA per wound, Tegaderm film was applied to protect the wound site.

### 2.13. In Vivo Wound Healing Study

After infection on day −1, 0.5 mg of GSNO, an equivalent amount of NO-loaded NC-GSNO (2.5 mg), and an amount of NC (2.5 mg) corresponding to the total mass of NC-GSNO were topically applied starting from day 0 at 2-day intervals, followed by wound area measurements. On the final day of the experiment, all the mice were photographed and euthanized using CO_2_ inhalation. Wound tissues (2 cm^2^ per wound) were excised and transferred to fresh PBS (pH 7.4) for an in vivo wound CFU analysis. The tissues were minced and sonicated for 5 min, followed by filtration through a 70 µm cell strainer to remove tissue debris. The resulting suspensions were serially diluted and plated for CFU enumeration.

### 2.14. Histology

After the final in vivo wound healing study, all the mice were euthanized using CO_2_. The wound tissues were excised and transferred to formalin for 12 h. After fixation, the tissues were dehydrated through a graded ethanol series and embedded in paraffin blocks. Sections (5 µm thickness) were obtained using a microtome (Shandon, Pittsburgh, PA, USA). Hematoxylin and eosin (H&E), Masson’s trichrome, and Twort’s Gram staining were conducted following the manufacturers’ protocols.

### 2.15. Statistics

All statistical analyses were performed using a one- or two-way analysis of variance followed by Dunnett’s multiple comparison post hoc test in GraphPad Prism 8 (GraphPad Software, Inc., La Jolla, CA, USA). Data are presented as the mean ± standard deviation (SD), and *p*-values < 0.05 were considered statistically significant.

## 3. Results

### 3.1. Characterization of NC-GSNO

The synthesis of NC-GSNO was performed in two separate steps (Figure 1A). First, an NHS ester was formed using EDC/NHS in an aqueous suspension of commercially purchased nanocellulose obtained by TEMPO oxidation (NC). After washing to remove the residual byproducts, the NC-NHS ester was resuspended and reacted with the primary amine of GSNO to form an amide linkage. The resulting product was light pink in color, originating from the dark pink color of GSNO.

To confirm the conjugation of GSNO to NC, an ^1^H-NMR analysis was performed (Figure 1B). The spectra showed peaks corresponding to GSNO, including the α-protons of cysteine (a, 4.68–4.70 ppm) and glycine (b, 4.12–4.14 ppm), as well as the H1 proton of NC (1, 4.54 ppm), indicating successful conjugation. An FTIR analysis further confirmed the presence of GSNO, with the amide I (C=O) band of GSNO observed at 1640 cm^−1^ (shifted from 1650 cm^−1^) [43] and the C–O–C linkages of NC at 1030 cm^−1^ (Figure 1C) [44]. The GSNO and NO loadings of NC-GSNO were calculated from the AUC obtained using a Sievers Nitric Oxide Analyzer and were found to be 20 ± 3% and 0.59 ± 0.08 µmol/mg, respectively (*n* = 3).

A Griess reaction was performed to confirm the presence of NO in NC-GSNO. When released NO is converted to nitrite (NO_2_^−^), the Griess reagent produces a magenta-colored compound with a maximum UV absorption at 548 nm. In the presence of the Griess reagent, NC-GSNO showed a conspicuous increase in UV absorption at 548 nm (Figure 1D). Because the S–NO bond of GSNO exhibits maximum UV absorbance at 335 nm, concentration-dependent UV absorption profiles were analyzed, showing a concentration-dependent increase at 335 nm (Figure 1E). Collectively, these results indicated that GSNO had successfully been conjugated to NC.

### 3.2. Hydrogel Properties of NC-GSNO

**Figure 2:**
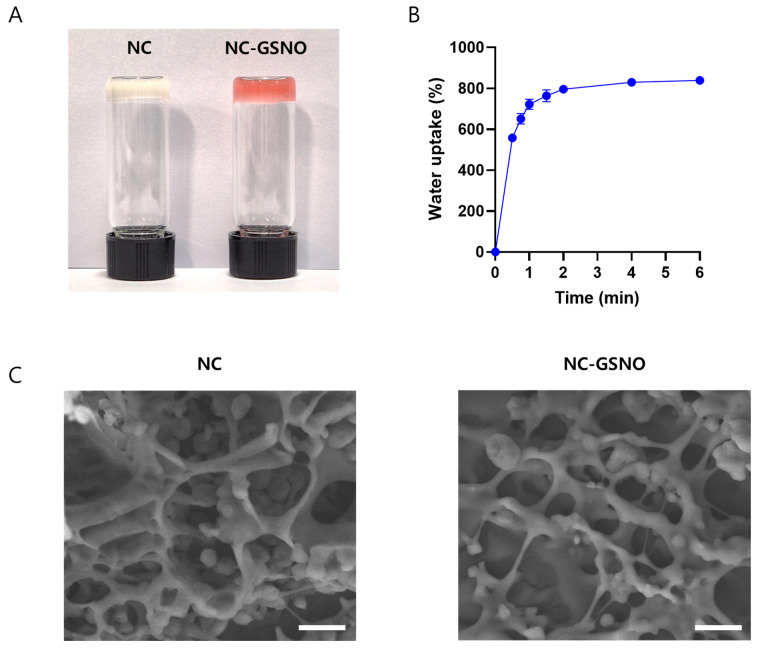
Hydrogel properties of NC-GSNO: (**A**) Image of inverted vials of the NC and NC-GSNO hydrogels. (**B**) Water uptake analysis of NC-GSNO (*n* = 3). (**C**) SEM images of NC and NC-GSNO. The scale bar represents 1 µm. Data are represented as the mean ± SD.

NC is known to form 3D network structures capable of hydrogel formation. As shown in the inverted vial image (Figure 2A), both the NC and NC-GSNO hydrogels retained their shapes, indicating successful gel formation. The water uptake test revealed rapid absorption behavior, reaching near equilibrium within 5–6 min and achieving up to 800% water uptake, suggesting high porosity and hydrophilicity (Figure 2B). In addition, SEM images showed an interconnected 3D porous structure for both hydrogels, which provided abundant water channels and structural stability (Figure 2C). These results collectively confirmed that NC-GSNO preserved the hydrogel-forming ability of the NC while maintaining a similar 3D network morphology.

### 3.3. GSNO Leakage and NO Release Study

**Figure 3:**
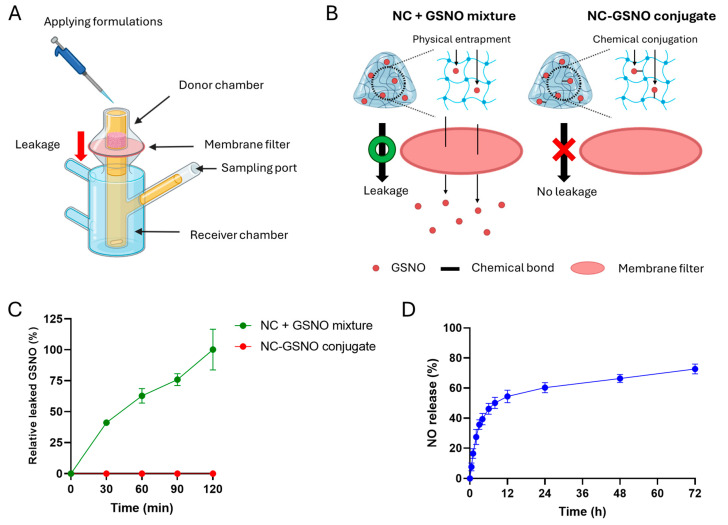
GSNO leakage and NO release study: (**A**) Schematic of the GSNO leakage study using the Franz diffusion cell. (**B**) Schematic of GSNO from the physical mixture of NC + GSNO and the NC-GSNO conjugated hydrogel. (**C**) HPLC quantification analysis of leaked GSNO in the receiver chamber (*n* = 3). The data were normalized to the NC + GSNO group at 120 min, which was set to 100%. (**D**) NO release profile of NC-GSNO at 37 °C (*n* = 3). The data are presented as the mean ± SD.

To further confirm that GSNO was effectively conjugated to the NC, a Franz diffusion cell analysis was performed (Figure 3A). In the NC + GSNO mixture group, in which no chemical conjugation occurred and GSNO was physically entrapped within the NC hydrogel scaffolds, rapid GSNO leakage was observed owing to diffusion (Figure 3B). In contrast, no significant GSNO leakage occurred in the NC-GSNO group owing to the strong chemical conjugation. HPLC quantification of the receiver chamber showed rapid GSNO leakage in the NC + GSNO mixture group, owing to the simple physical GSNO loading via electrostatic interactions (Figure 3C). However, in the NC-GSNO group, negligible GSNO was detected over the entire 120 min analysis, confirming the robust chemical conjugation between NC and GSNO. These results demonstrate that NC-GSNO had a much higher GSNO retention ability than the simple physical mixture of NC + GSNO. Furthermore, conjugated GSNO exhibited initial burst NO release, followed by sustained NO release over 72 h (Figure 3D). Collectively, these findings indicate that NC-GSNO effectively retains GSNO within its hydrogel scaffold, preventing leakage and enabling controlled NO release that may further facilitate healing of infected wounds.

### 3.4. In Vitro Antibacterial Study

**Figure 4:**
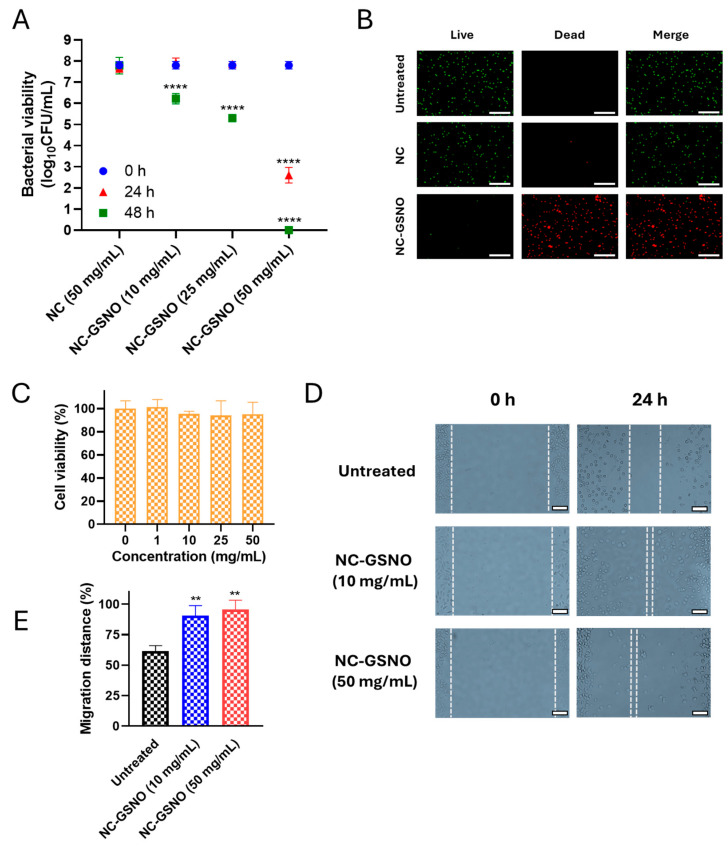
In vitro antibacterial and cell migration study: (**A**) In vitro antibacterial study of NC-GSNO (10, 25, and 50 mg/mL) and NC (50 mg/mL) after 24 h and 48 h incubation (*n* = 3). The data are independent and presented as the mean ± SD, analyzed by two-way ANOVA followed by Dunnett’s post hoc test (95% confidence level, compared with 0 h of each group). (**B**) Live/Dead assay of NC-GSNO after exposure for 48 h at a 50 mg/mL concentration. The scale bar represents 50 µm. (**C**) Cytotoxicity assay (CCK-8) of NC-GSNO at various concentrations (0, 1, 10, 25, and 50 mg/mL) after 24 h incubation (*n* = 3). Cell viability was normalized to the untreated control (100%). (**D**) Representative images (scale bar 100 µm, dotted lines denote the migration range) and (**E**) quantification analysis of fibroblast migration at 24 h after treatment with NC-GSNO at a concentration 10 and 50 mg/mL respectively (*n* = 3). The data are independent and presented as the mean ± SD, analyzed by one-way ANOVA followed by Dunnett’s post hoc test (95% confidence level). Significance levels: *p* < 0.01 (**), *p* < 0.0001 (****) compared with the untreated group.

Prolonged exposure to high concentrations of NO is crucial for efficient wound healing and MRSA eradication. To elucidate the extent of the exposure-dependent antibacterial effects of NC-GSNO, its concentration- and time-dependent antibacterial effects were determined through in vitro antibacterial experiments (Figure 4A). Regarding concentration dependence, after 24 h of incubation, NC-GSNO showed no significant CFU reduction up to 25 mg/mL, whereas a 5-log reduction was observed at 50 mg/mL. In the 48 h incubation model, gradual CFU reductions of 2-log at 10 mg/mL, 3-log at 25 mg/mL, and 8-log (near complete eradication of MRSA) at 50 mg/mL were observed. The NC group (50 mg/mL) showed no significant bactericidal effects after 24 or 48 h. These results indicate that sustained NO release of NC-GSNO at 50 mg/mL after 48 h contributes to effective antibacterial activity.

To further demonstrate the antibacterial effects, a Live/Dead assay was conducted (Figure 4B). In this assay, live cells emit green SYTO-9 fluorescence, whereas dead cells emit red PI fluorescence. NC and NC-GSNO at a concentration of 50 mg/mL were incubated for 48 h (Figure 4A). A strong red signal was observed in the NC-GSNO group, whereas green fluorescence was predominant in the NC and untreated groups. This indicates that nearly all MRSA was eradicated in the NC-GSNO group (50 mg/mL) owing to the effective cumulative nitrosative stress from sustained NO release.

### 3.5. In Vitro Cytotoxicity Study

The cytotoxicity of NC-GSNO was evaluated using a CCK-8 assay with L929 murine fibroblasts. After 24 h of incubation, NC-GSNO did not induce significant cytotoxicity, and cell viability remained above 90% at concentrations up to 50 mg/mL (Figure 4C).

### 3.6. In Vitro Cell Migration Study

An in vitro cell migration assay was performed to evaluate the effects of NO on fibroblast migration at a concentration of 10 and 50 mg/mL (Figure 4D). After 24 h, cells treated with NC-GSNO at 10 mg/mL and 50 mg/mL showed nearly complete migration across the scratch gap (90 ± 8%, 95 ± 8%, respectively), whereas the untreated group exhibited only partial migration (61 ± 4%) (Figure 4E). These results indicate that NC-GSNO significantly enhances fibroblast migration, a crucial process for effective wound repair. Overall, these findings demonstrate the beneficial potential of NC-GSNO for wound treatment.

### 3.7. In Vivo Wound Healing Study

**Figure 5:**
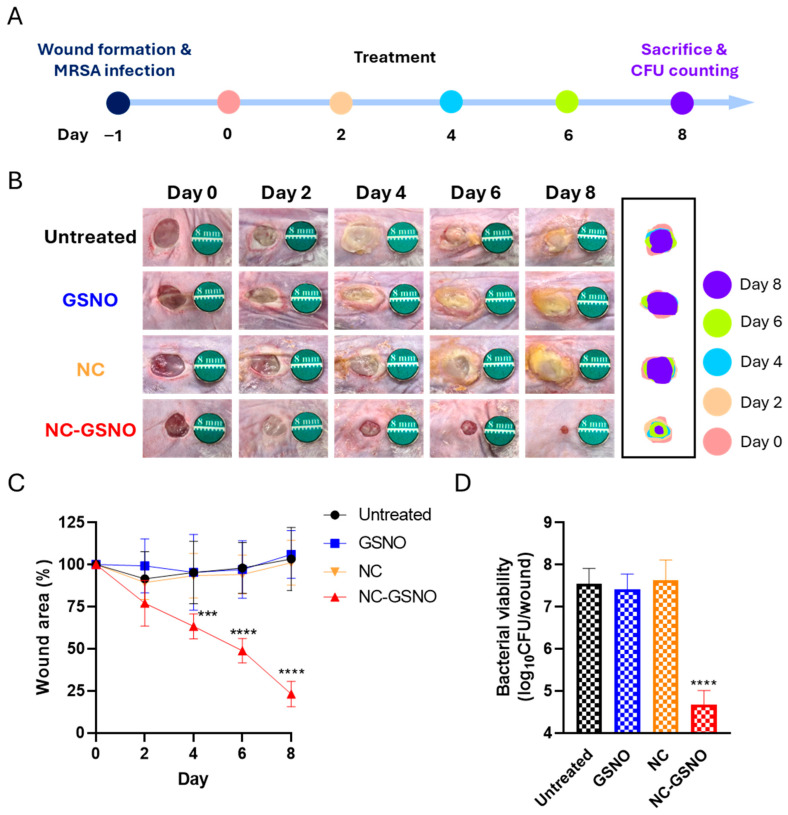
In vivo wound healing study: (**A**) Schematic of the in vivo wound healing study. (**B**) Images of wound healing across 8 days with green marker (8 mm diameter) for objective wound area comparison. The wound areas of each group were expressed by 2-day intervals. (**C**) Wound area reduction profiles in the untreated, GSNO-, NC-, and NC-GSNO-treated groups (*n* = 5). The data are biologically independent and presented as the mean ± SD using two-way ANOVA followed by Dunnett’s post hoc test (95% confidence level). (**D**) CFU of MRSA in wound tissues on day 8 (*n* = 3). The data are biologically independent and presented as the mean ± SD using one-way ANOVA followed by Dunnett’s post hoc test (95% confidence level). Significance level: *p* < 0.001 (***), *p* < 0.0001 (****) compared with the untreated group.

To evaluate the effects of NC-GSNO on infected-wound healing, mice were treated for 2 days, and their wound sizes were monitored for 8 days (Figure 5A). The wound morphology in the untreated, GSNO-treated, and NC-treated groups exhibited characteristic features of biofilm formation, including yellowish coloration, slimy exudate layers, and dense surface aggregates, with no conspicuous reductions in the wound areas over the 8-day period. In contrast, the NC-GSNO-treated group showed substantially reduced biofilm coverage and a gradual decrease in wound area (Figure 5B). Quantitative analyses of the wound areas revealed that the untreated, GSNO-treated, and NC-treated groups maintained wound areas similar to those at the initial measurement throughout the 8 days, likely due to chronic inflammation caused by the MRSA infection. The NC-GSNO-treated group, however, showed progressive wound closure, with a 25% reduction on day 2, 50% reduction on day 6, and 75% reduction on day 8, reflecting effective MRSA eradication and the facilitated wound healing effects of NO (Figure 5C). To further confirm MRSA eradication at the wound site, an in vivo CFU analysis was performed on day 8 after euthanizing the mice and excising the wound tissues. The results showed a significant 3-log reduction in the bacterial burden in the NC-GSNO-treated group compared to that in the untreated, GSNO-treated, and NC-treated groups (Figure 5D). These findings indicate that NC-GSNO effectively exerted antibacterial effects in MRSA-infected full-thickness wounds, thereby promoting wound healing.

### 3.8. Histology

**Figure 6:**
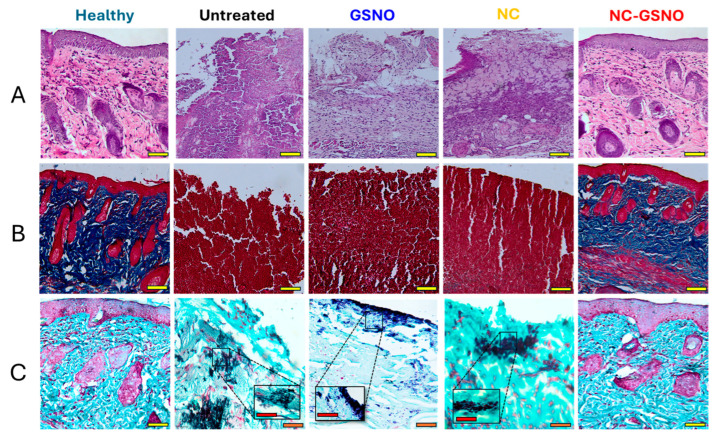
Representative histological images of wound tissue from the healthy, untreated, GSNO, NC, and NC-GSNO groups: (**A**) Hematoxylin and eosin staining. (**B**) Trichrome staining. (**C**) Twort’s Gram staining of wound tissue. Scale bar = 100 µm for 20× (yellow), 50 µm for 40× (orange), and 10 µm for further magnification (red).

The wound-healing effect was further confirmed by a histological assessment. H&E staining revealed the full recovery of hair follicles, sebaceous glands, and epidermal barriers in the NC-GSNO-treated group (Figure 6A). In contrast, no distinct epidermis or hair follicles with sebaceous glands were observed in the untreated, GSNO-, and NC-treated groups, likely due to the continuous inflammation caused by the MRSA infection. This persistent inflammation also led to delayed collagen regeneration in dermal areas, as observed following Masson’s trichrome staining (Figure 6B). Violet-colored regions, indicating collagen deposition in the dermis, were rarely observed in the untreated, GSNO-, and NC-treated groups, whereas distinct violet regions were evident in the healthy and NC-GSNO-treated groups, indicating the full recovery of collagen in the dermis. Finally, Twort’s Gram staining was performed to confirm the presence of MRSA in the wound area (Figure 6C). Dark-violet MRSA colonies were observed in the untreated, GSNO-, and NC-treated groups, whereas no MRSA was detected in the healthy and NC-GSNO-treated group, indicating effective bacterial eradication at the wound site.

## 4. Discussion

Cutaneous wound infections with MRSA remain major clinical challenges due to the antibiotic resistance of MRSA and poor wound healing outcomes [1,2]. The continuous NO exposure at the wound site is crucial for bacterial eradication and tissue regeneration [16]. Most of the previous GSNO formulations, including both simple loading and conjugation approaches, have been limited by one or more limitations—such as GSNO leakage, low NO loading, burst NO release, use of harsh external stimuli, and complex multistep fabrication processes—which ultimately compromise their therapeutic efficacy [19,20,21,22,25,26,27]. To address these issues, we developed GSNO-conjugated TEMPO-oxidized NC (NC-GSNO) using a simple and efficient GSNO conjugation strategy. The resulting NC–GSNO hydrogel achieved minimal leakage with high GSNO loading and exhibited sustained NO release without the use of harsh external stimuli, thereby effectively overcoming the limitations associated with physically mixed and other conjugated GSNO systems. This system represents a promising platform for enhanced healing of MRSA-infected wounds.

In this study, nanocellulose fibers obtained by TEMPO oxidation (NC) were selected as a platform material due to its fibrous structure and high density of surface carboxyl groups, which facilitate efficient chemical functionalization [28]. The design strategy was to exploit these reactive carboxylic groups to conjugate GSNO via stable amide bond formation, thereby immobilizing the NO donor within the cellulose network. A similar conjugation strategy has been previously reported [25]. In the present system, optimized EDC/NHS chemistry was employed to synthesize the NC-GSNO conjugate (Figure 1A). This allowed the primary amine of GSNO to react efficiently with multiple carboxyl groups on NC, forming stable NC-GSNO conjugates. An FTIR analysis of NC-GSNO confirmed the conjugation, showing the presence of amide bonds from GSNO and ether linkages from NC (Figure 1B). A proton NMR analysis also revealed the presence of the distinct alpha protons of GSNO and H1 proton of NC (Figure 1C). UV absorbance spectra also revealed the presence of NO in NC-GSNO through the Griess reaction at 548 nm and a concentration-dependent absorption profile at 335 nm (Figure 1D,E). The NO loading capacity was 0.59 µmol/mg, which was more than 8 times that of previous research, demonstrating efficient GSNO conjugation [25]. The SEM image of NC-GSNO revealed a highly porous structure typical of hydrogels formed by 3D polymer networks upon water absorption (Figure 2C). This porosity was similar to that of the NC, indicating that the characteristic hydrogel structure was preserved after GSNO conjugation. Owing to its robust 3D hydrogel network, the NC-GSNO hydrogel is expected to be retained on wound tissue and biofilms potentially facilitating prolonged contact and sustained NO delivery at the wound site.

In vitro GSNO leakage tests using a Franz diffusion cell revealed that the physically mixed NC + GSNO hydrogel allowed rapid GSNO leakage through the membrane, whereas the conjugated NC-GSNO hydrogel showed no detectable leakage (Figure 3A–C). The release study revealed an initial burst of NO, followed by sustained NO release for more than 72 h (Figure 3D). The initial burst release of NO is likely attributed to the rapid decomposition of GSNO molecules located near the surface of the hydrogel upon immediate hydration. In contrast, the subsequent sustained NO release may be attributed to the hydrophilic microenvironment of the NC hydrogel, where numerous hydroxyl and carboxyl groups stabilize the S–N=O bond. Indeed, microenvironmental factors that provide hydrogen bonds have been suggested to stabilize the S–N=O bond, thereby improving its stability [32,33]. For example, previous studies have reported significantly enhanced sustained NO release in hydrophilic formulations that provide multiple hydrogen bonds [34,35]. Taken together, these results demonstrate that the conjugated GSNO in NC was firmly retained within the hydrogel structure, allowing an initial burst followed by sustained NO release owing to the stabilization of the S–N=O bond.

In the in vitro antibacterial study, the bactericidal effect was both concentration- and time-dependent, with a progressive increase in bacterial eradication over time (Figure 4A,B). This phenomenon is consistent with that in previous studies showing that the antibacterial mechanism of NO depends on both concentration and duration [45,46]. Notably, NC-GSNO exhibited an initial burst release followed by sustained NO release, providing both rapid early-phase exposure and prolonged antibacterial activity. Mechanistically, NO exerts antimicrobial activity through direct nitrosative/oxidative modifications of proteins, lipids and DNA; formation of reactive nitrogen species such as peroxynitrite; and disruption of respiratory enzymes. These actions collectively contribute to bacterial death. Therefore, the early high local NO concentration promotes rapid bacterial killing, while sustained release degrades residual biofilm structures and prevents bacterial regrowth and biofilm recovery [47,48]. These results highlight the importance of achieving a high local NO concentration at the initial stage and maintaining sustained NO release, which is favorable for effective antibacterial effects at the wound site. In vitro cytotoxicity study showed no significant toxicity up to 50 mg/mL (Figure 4C). Cell migration studies demonstrated that NC-GSNO significantly enhanced fibroblast migration at the non-toxic concentrations (Figure 4E). It is well established that during wound healing, low concentrations of NO facilitate tissue regeneration by promoting both essential cell migration and the proliferation of keratinocytes, fibroblasts, and endothelial cells [49]. Our observed effect can therefore be attributed to the sustained NO release profile of NC-GSNO [50].

In the in vivo infected-wound study, the NC-treated and untreated groups showed chronic wounds for 8 days without a reduction in wound size, owing to severe MRSA infection-induced inflammation (Figure 5A–C). Because of rapid clearance in the GSNO group, NO release was not sufficient for MRSA eradication, resulting in morphologies similar to those in the untreated group. However, NC-GSNO showed conspicuous rapid wound healing and a reduction in the wound size to 25% of the initial size within 8 days without biofilm formation and a reduced bacterial burden that was 3-log less than that in the untreated group (Figure 5D). These results can be attributed to the combined antibacterial and pro-migratory effects produced by the initial burst and subsequent sustained NO release of the highly NO-loaded NC-GSNO hydrogel. This release profile prevented GSNO leakage and provided continuous NO exposure to the wound tissue and biofilms, thereby enhancing wound healing.

H&E staining showed well-recovered epidermal and dermal areas in the NC-GSNO-treated group; however, these observations were not made in the GSNO-treated, NC-treated, and untreated groups (Figure 6A). Trichrome staining showed collagen regeneration, which was clearly observed in the NC-GSNO-treated group but not in the GSNO-treated, NC-treated, and untreated groups (Figure 6B). Twort’s Gram staining showed the presence of MRSA in the NC- and GSNO-treated and untreated groups (Figure 6C). Untreated MRSA infections exacerbated inflammation, leading to delayed wound closure and chronic wounds. In contrast, no MRSA was observed in the NC-GSNO-treated group due to the antibacterial effects of NC-GSNO, which led to rapid wound closure and tissue recovery. In summary, the NC-GSNO hydrogel showed potent wound healing in the MRSA-infected wound model, potentially due to its retentive nature, with sustained NO release characteristics following GSNO conjugation. It should be noted, however, that the retention of NC-GSNO at the wound site was inferred from the generally recognized retention properties of hydrogel-based materials rather than directly verified in this study [51,52,53]. Future work using imaging or retention assays could provide more direct evidence of this behavior and clarify its contribution to the sustained therapeutic effect.

## 5. Conclusions

In this study, NC-GSNO was synthesized using simple EDC/NHS chemistry for the effective treatment of MRSA-infected cutaneous wounds. Although GSNO-conjugated systems have been developed to prevent GSNO leakage observed in physically GSNO-loaded systems, most previously reported GSNO-conjugated strategies still suffer from one or more of the following limitations: low NO loading, rapid NO release, use of harsh external stimuli, and complex multistep fabrication processes that may ultimately limit their therapeutic efficacy. To address these limitations, NC hydrogel was employed for GSNO conjugation through a simple and efficient process, enabling high NO loading and sustained NO release while minimizing leakage. In vitro, NC-GSNO exhibited NO-dependent bactericidal effects and enhanced fibroblast migration, and in vivo, it improved wound healing in MRSA-infected mice. Collectively, these findings demonstrate that NC-GSNO provides a biocompatible and controlled NO-delivery platform that releases NO sustainably without the use of harsh external stimuli, minimizes systemic leakage and associated side effects, and holds promise as a therapeutic platform for MRSA-infected wounds.

## Data Availability

The original contributions presented in the study are included in the article, and further inquiries can be directed to the corresponding author.
